# Hydroxynitrile Lyases with α/β-Hydrolase Fold: Two Enzymes with Almost Identical 3D Structures but Opposite Enantioselectivities and Different Reaction Mechanisms

**DOI:** 10.1002/cbic.201200239

**Published:** 2012-07-31

**Authors:** Jennifer N Andexer, Nicole Staunig, Thorsten Eggert, Christoph Kratky, Martina Pohl, Karl Gruber

**Affiliations:** [a] Institute of Pharmaceutical Sciences, Albert-Ludwigs-University FreiburgAlbertstrasse 25, 79104 Freiburg (Germany); [b] Institute of Molecular Biosciences, University of GrazHumboldtstrasse 50/3, 8010 Graz (Austria) E-mail: karl.gruber@uni-graz.at; [c] Evocatal GmbHMerowingerplatz 1a, 40225 Düsseldorf (Germany); [d] IBG-1: Biotechnology, Forschungszentrum Jülich GmbH52425 Jülich (Germany)

**Keywords:** biocatalysis, C–C lyase, enzyme mechanisms, molecular modeling, X-ray crystallography

## Abstract

Hydroxynitrile lyases (HNLs) catalyze the cleavage of cyanohydrins to yield hydrocyanic acid (HCN) and the respective carbonyl compound and are key enzymes in the process of cyanogenesis in plants. In organic syntheses, HNLs are used as biocatalysts for the formation of enantiopure cyanohydrins. We determined the structure of the recently identified, *R*-selective HNL from *Arabidopsis thaliana* (*At*HNL) at a crystallographic resolution of 2.5 Å. The structure exhibits an α/β-hydrolase fold, very similar to the homologous, but *S*-selective, HNL from *Hevea brasiliensis* (*Hb*HNL). The similarities also extend to the active sites of these enzymes, with a Ser-His-Asp catalytic triad present in all three cases. In order to elucidate the mode of substrate binding and to understand the unexpected opposite enantioselectivity of *At*HNL, complexes of the enzyme with both (*R*)- and (*S*)-mandelonitrile were modeled using molecular docking simulations. Compared to the complex of *Hb*HNL with (*S*)-mandelonitrile, the calculations produced an approximate mirror image binding mode of the substrate with the phenyl rings located at very similar positions, but with the cyano groups pointing in opposite directions. A catalytic mechanism for *At*HNL is proposed, in which His236 from the catalytic triad acts as a general base and the emerging negative charge on the cyano group is stabilized by main-chain amide groups and an α-helix dipole very similar to α/β-hydrolases. This mechanistic proposal is additionally supported by mutagenesis studies.

## Introduction

**Scheme 1:**
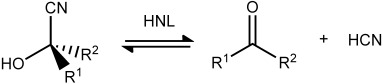
HNL-catalyzed cleavage and synthesis of chiral cyanohydrins.

Hydroxynitrile lyases (HNLs) catalyze the cleavage of cyanohydrins into the corresponding carbonyl compound and hydrocyanic acid (Scheme 1). In plants, the HNL-catalyzed reaction is part of a defense mechanism against herbivores and microorganisms.^1^ Since the natural HNL substrates are small and often achiral cyanohydrins (e.g., acetone cyanohydrin), the development of enantioselectivity has not been the subject of evolutionary pressure. Nevertheless, most HNLs show pronounced enantioselectivity when exposed to non-natural chiral substrates. Thus, the stereoselective C–C-bond formation by the HNL-catalyzed addition of HCN to aldehydes or prochiral ketones (i.e., the reverse of the biological reaction) constitutes an industrially exploited route to produce enantiopure cyanohydrins as building blocks for the synthesis of pharmaceuticals and agrochemicals.^2^

HNLs form a diverse group of enzymes, which appear to have evolved through convergent evolution.2d, 3 To date, at least five different enzyme classes have been identified: the FAD-dependent (*R*)-HNLs from various *Rosaceae* being related to glucose-methanol-choline oxidoreductases;^4^ the *R*-selective enzyme from *Linum usitatissimum* showing homology to zinc-dependent alcohol-dehydrogenases;^5^ the serine-carboxypeptidase-like (*S*)*-*HNL from *Sorghum bicolor*;^6^ the *S-*selective enzymes from *Hevea brasiliensis* (para rubber tree, *Hb*HNL),^7^ from *Manihot esculenta* (cassava, *Me*HNL)^8^ and from *Baliospermum montanum*,^9^ exhibiting an α/β-hydrolase fold;^10^ and most recently two HNLs from endophytic bacteria showing similarities to members of the cupin superfamily of proteins.^11^

*Hb*HNL and *Me*HNL are among the best characterized HNLs. They are so similar in sequence (77 % identity), 3D structure (root-mean-square deviations (RMSD) of 0.3 Å for a superposition of 230 out of 256 Cα-atoms) and biochemical properties that we used the (somewhat better characterized) *Hb*HNL as prototype for (*S*)-HNLs with an α/β-hydrolase fold. Their enzymatic mechanism involves the Ser-His-Asp catalytic triad as a general base deprotonating the cyanohydrin substrate with the histidine residue being the actual base and the serine hydroxyl group acting as a mediator.^12^ The necessity of a general base in HNL catalysis has been proposed more than 45 years ago by Becker and Pfeil,^13^ who also predicted the requirement for a positive charge in the active sites of HNLs to stabilize the negative charge evolving at the cyano group during the reaction. For *Hb*HNL, this role was unequivocally ascribed to Lys236 based on structural and mutagenesis data.12a

In 2007 a hydroxynitrile lyase from the noncyanogenic plant *Arabidopsis thaliana* (mouse-ear cress) was discovered (*At*HNL) which exhibits high sequence similarity to the *S*-selective *Hb*HNL (45 % identity and 67 % similarity) but quite unexpectedly was found to be *R*-selective for a wide range of converted cyanohydrins.^14^ Currently the natural function of this enzyme remains unknown; however *At*HNL exhibits equal kinetic parameters concerning the synthesis and cleavage of mandelonitrile as *Me*HNL.^15^ Furthermore, *At*HNL is also a useful catalyst for the synthesis of β-amino alcohols.^16^ The lower stability of *At*HNL in the acidic pH range has recently been successfully improved by immobilization^17^ and site-directed mutagenesis.^18^

Previously, *S* selectivity has been considered a characteristic feature of HNLs from the α/β-hydrolase family, and *At*HNL is thus the first *R*-selective member of this fold family. The Ser-His-Asp catalytic triad is conserved in *At*HNL and is crucial for catalysis,^14^ whereas *Hb*HNL and *At*HNL differ with respect to several other residues surrounding the active site. Specifically, the lysine residue crucial for stabilizing the negative charge on the cyanide (Lys236 in *Hb*HNL) is replaced by methionine. Homology studies did not propose a residue with a positive charge in the immediate neighborhood of the active site to take over the lysine’s function in catalysis.

We have earlier speculated about the mechanism of *At*HNL based on a homology model.^14^ These ideas can now be refined and partially revised based on the X-ray crystal structure analysis of the native enzyme, which is discussed here together with modeling calculations and mutagenesis studies to address the following questions:

What is the molecular basis for the inverse enantioselectivity of *At*HNL with respect to the other known α/β-hydrolase HNLs, despite their significant structural and sequence similarity?

What is the reaction mechanism of *At*HNL, specifically how is the negative charge of the nascent cyanide ion stabilized?

## Results

### Overall structure

**Figure 1:**
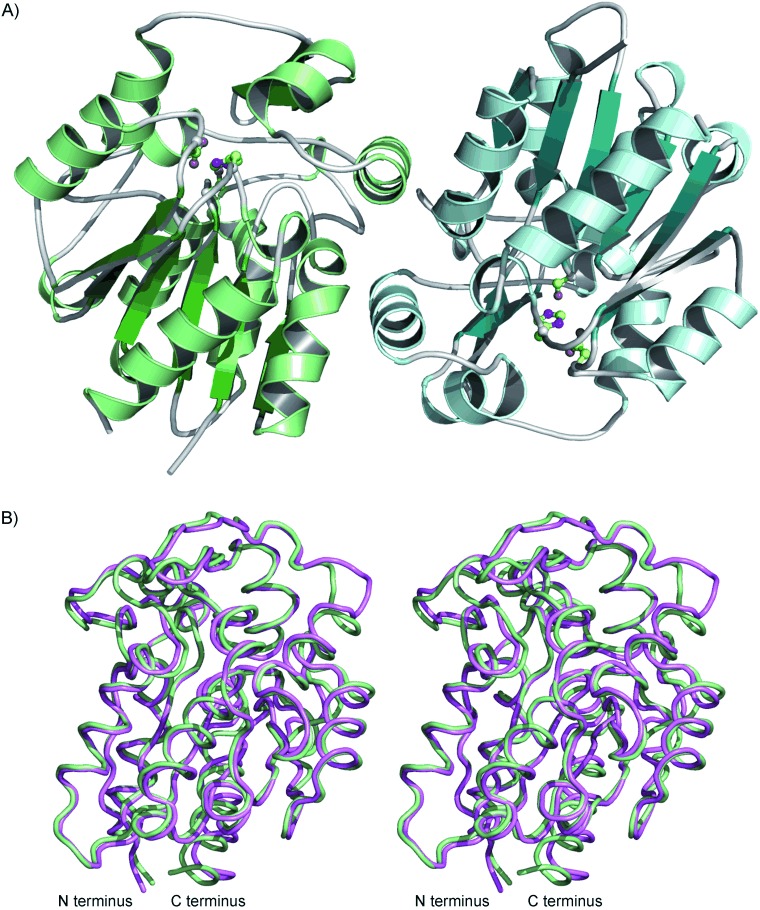
Overall structure. A) Schematic representation of the dimer of *At*HNL. The subunits are colored in green and cyan respectively. Amino acid residues forming the catalytic triad (Ser81, His236 and Asp208) are shown with a ball-and-stick representation. B) Stereoscopic representation of the superposition of the structures of *At*HNL (magenta) and of *Hb*HNL (cyan). Figures 1 and 2 were prepared using the program PyMOL (http://www.pymol.org).

We report the crystal structure at 2.5 Å resolution (Table 1) of the HNL from *A. thaliana,* for which sequence similarities and mutational data for putative active-site residues^14^ had suggested significant structural similarity with the known HNLs with α/β-hydrolase folds. This prediction is clearly confirmed by the crystal structure (Figure 1 A). With an root-mean-square deviation of 0.8 Å (for the superposition of 237 out of 258 Cα-atoms) the structure of *At*HNL is indeed very similar to that of the HNL from *H. brasiliensis* (*Hb*HNL, PDB ID: 1QJ4). The only significant fold difference between the two structures is observed in a loop at the entrance of the active site (Figure 1 B). The overall structure is also very similar to the previously described homology model of the enzyme^14^ (Figure S1).

**Table 1:** Summary of crystallographic data

	*At*HNL
X-ray source	EMBL-X13
wavelength [Å]	0.8081
temperature [K]	100
space group	*P*2_1_
cell parameters *a* [Å] *b* [Å] *c* [Å] *β* [°]	50.25 223.31 50.20 101.5
resolution range (outer shell)	25.0–2.5 (2.56–2.50)
*R*_sym_	0.071 (0.210)
*I*/*σ* (*I*)	18.9 (4.8)
completeness [%]	89.6 (87.5)
redundancy	3.4 (2.9)
unique reflections	33 106
*R*/*R*_free_ [%]	15.9/21.0
RMSD from ideality bond lengths [å] bond angles [°] dihedral angles [°] planarity [Å]	0.006 0.9 16.6 0.004
average B factors protein water	28.0 15.8
PDB ID	3DQZ

The asymmetric unit consists of four protein molecules forming two independent dimers (Figure 1 A). Analyses of the interaction surfaces using the protein interactions, surfaces and assemblies (PISA) server^19^ yielded interface areas of approximately 870 Å^2^ in both dimers. Contacts between the two protein chains are mostly hydrophobic in nature but also include a salt-bridge interaction between Lys24 of one molecule and Glu165 of the other. The PISA-analysis on the interaction surfaces indicates that the dimeric arrangement is also likely to be present in solution. In fact, this was verified by size-exclusion chromatography.^15^ Similarly, the crystal structure of *Me*HNL also contains dimers in the respective asymmetric units,^8^ whereas a single polypeptide chain forms the asymmetric unit in the *Hb*HNL crystal structure.^7^ In the latter case, however, corresponding dimers are generated through the application of crystallographic symmetry transformations. These *Hb*HNL dimers were also shown experimentally to persist in solution.^20^

### Substrate binding

Docking calculations were carried out using AutoDock v4^21^ in order to elucidate the mode of substrate binding. These calculations identified low-energy binding modes of (*R*)- and (*S*)-mandelonitrile to the active site of *At*HNL and produced a single cluster of binding modes in each case.

**Figure 2:**
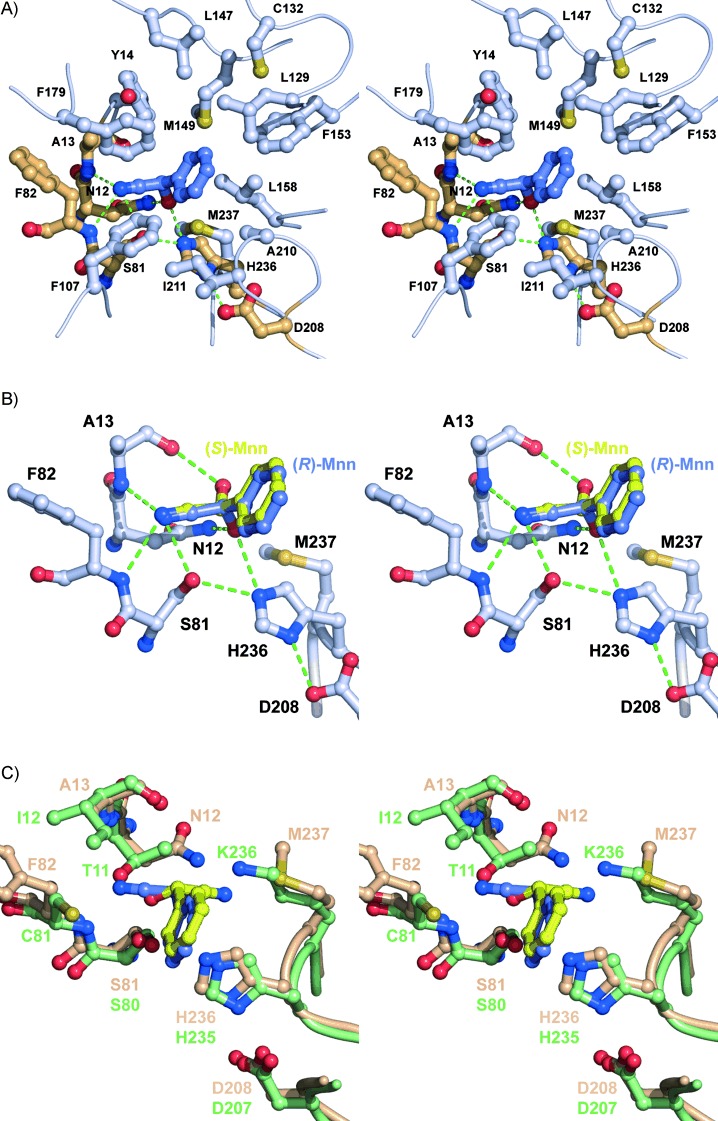
Structures of substrate complexes. A) Stereoscopic representation of the modeled complex of *At*HNL with (*R*)-mandelonitrile. Amino acid residues of the catalytic triad (Ser81, His236 and Asp208) as well as residues forming polar interactions with the bound substrate are shown in orange, amino acid residues which build up the mostly hydrophobic pocket housing the phenyl ring of the substrate are shown in white. Green dashed lines signify possible hydrogen bonding interactions. B) Stereoscopic representation of the superposition of the modeled complexes of *At*HNL with (*R*)- (blue) and (*S*)-mandelonitrile (yellow). C) Stereoscopic representation of the superposition of modeled complex of *At*HNL (orange) with (*R*)-mandelonitrile (blue) and the experimentally determined complex of *Hb*HNL (light green) with (*S*)-mandelonitrile (yellow).^24^

In the modeled complex with (*R*)-mandelonitrile (Figure 2 A) the hydroxyl group of the substrate is hydrogen bonded to His236, and also interacts with the amide group of Asn12. The cyano group is oriented toward the main-chain NH-groups of Phe82 and Ala13 and is located at the N-terminal end of the α-helix following the “nucleophile elbow” in the α/β-hydrolase fold.^10^ The phenyl group is buried in a predominantly hydrophobic pocket formed by phenylalanine, tyrosine, methionine, leucine and isoleucine residues (Figure 2 A).

A structural superposition of the modeled complexes with (*R*)- and (*S*)-mandelonitrile revealed identical positions of the phenyl rings and the cyano groups respectively (Figure 2 B). In the case of the *S* enantiomer, however, there is no hydrogen bond between the substrate’s hydroxyl group and one of the residues of the catalytic triad. Instead, this group now interacts with the main-chain carbonyl oxygen of Ala13.

### Mutational analysis

Table 2 lists several mutants generated to study the effect of amino acid exchanges of residues potentially relevant for catalysis. While the mutation of residues forming the catalytic triad—yielding inactive enzyme for each of the three residues—was reported previously,^14^ our substrate modeling studies indicated the involvement of Asn12 in substrate binding. Replacement of this residue by threonine (the corresponding residue in *Hb*HNL) indeed yielded inactive enzyme. On the other hand, mutation of Met237 to leucine did not impair *At*HNL activity, whereas the equivalent mutation in *Hb*HNL (Lys236Leu) led to inactive enzyme.12a

**Table 2:** Residual activity of *At*HNL variants relative to the wild type enzyme. Initial rate activities were measured as described previously^14^

Residue in *At*HNL	Corresponding residue in *Hb*HNL	Variant (*At*HNL)	Residual activity [%]
Asn12	Thr11	Asn12Thr	<2
Met237	Lys236	Met237Lys	n.d.[b]
Met237	Lys236	Met237Leu	100
Asn12, Met237	Thr11, Lys236	Asn12Thr Met237Lys	n.d.[b]
Ser81[a]	Ser80	Ser81Ala	<2
Asp208[a]	Asp207	Asp208Asn	<2
His236[a]	His235	His236Phe	<2

[a] Data taken from Andexer et al.^14^

[b] Expression of the *At*HNL variant resulted in insoluble protein.

## Discussion

Hydroxynitrile lyases appear to have evolved convergently from different ancestral proteins. Members of four enzyme classes have been subjected to detailed analysis so far, yielding quite different catalytic mechanisms. However, each of them conforms to the mechanistic requirements postulated by Becker and Pfeil for HNL catalysis,^13^ albeit in radically different structural implementations.4a, 22

*At*HNL is the first known *R*-selective HNL with an α/β-hydrolase fold. So far, *S* selectivity was considered a characteristic property of HNLs from this family, which includes *Hb*HNL, *Me*HNL, *Sb*HNL and the recently identified enzyme from *B. montanum*.^9^ The crystal structure of *At*HNL shows a striking structural similarity between *AtHNL* and *Hb*HNL, both in the polypeptide fold (Figure 1 B) and with respect to the presence and location of crucial active site residues, for example, the Ser-His-Asp catalytic triad (Figure 2 C). This begs for a molecular explanation of *At*HNL’s observed *R* enantioselectivity, since the structurally highly similar *Hb*HNL is *S* selective, and for an analysis how the mechanistic requirements for HNL-activity (general base and positive charge)^13^ are structurally implemented in *At*HNL.

### Enzyme–substrate complexes

Elucidation of the 3D structure of enzyme–substrate complexes by X-ray crystallography has been notoriously difficult for HNLs for a number of practical reasons, notably the rapid destruction of enzyme crystals upon contact with the cyanohydrin substrate. This is probably a result of the high turnover number in combination with the release of a gaseous reaction product (HCN). Problems are aggravated by the poor water solubility of most cyanohydrins. Thus, experimental 3D data for enzyme–substrate complexes are so far only available for *Hb*HNL12a and *Pa*HNL.4c On the other hand, docking simulations appear to work extremely well for HNLs,4a, 23 most probably because in all cases studied so far, changes in the protein structure upon substrate binding are minute. Thus, the experimental complex structures of *Hb*HNL had previously been predicted within a few tenths of an Ångstrom.12a, 24 Modeled substrate complexes of HNLs were also successfully used as the basis for enzyme engineering.^25^ In the absence of experimental 3D data for enzyme substrate complexes of *At*HNL, we performed docking calculations—to which we ascribe a high level of confidence—with the two standard model substrates (*R*)- and (*S*)-mandelonitrile.

The two enantiomers bind very similarly to *At*HNL, with almost identical orientations and locations of phenyl rings and cyano groups (Figure 2 B). With the phenyl ring and cyano group fixed, the hydroxyl group of mandelonitrile has to point into a different direction for the two enantiomers, interacting with His236 and Asn12 for the *R* and with the main chain carbonyl oxygen of Ala13 for the *S* enantiomer.

An earlier model of the substrate complex of *At*HNL^14^ was based on the homology model of the enzyme structure and used the complex of *Hb*HNL with (*S*)-mandelonitrile^24^ as guidance. In this model, (*R*)-mandelonitrile is bound with its cyano group pointing towards Met237 (Figure S2), which corresponds to Lys236 in *Hb*HNL. We believe that our present model, which was generated using a less biased docking approach, is more reliable.

### Enantioselectivity of *At*HNL

Rationalization for the striking *R* enantioselectivity of *At*HNL readily emerges from Figure 2 B. If we assume that the substrate docking calculations correctly predicted the complex structures for the two mandelonitrile enantiomers, then any reaction mechanism (see below) involving deprotonation at the cyanohydrin hydroxyl group is more likely to act on the (*R*)-mandelonitrile than on the *S* enantiomer. While the *R* enantiomer has the hydroxyl group within hydrogen bonding distance to a potential catalytic base (His236), the *S* enantiomer interacts with a main-chain carbonyl oxygen, which is unlikely to deprotonate a hydroxyl group.

While a molecular explanation for *At*HNL’s enantioselectivity is thus straightforward and plausible, a comparison with the structurally similar (*S*)-*Hb*HNL is instructive, specifically to analyze the molecular reasons for the observed inversion of enantioselectivity. Comparison of the reactive complex structures between *At*HNL and *Hb*HNL (Figure 2 C) shows that the residues of the catalytic triad and the substrates’ phenyl rings superimpose very well between *At*HNL and *Hb*HNL, while the substrates’ cyano groups point into opposite directions, interacting with Lys236 in *Hb*HNL12a, 24 and with the main-chain NH-groups of Phe82 and Ala13 in *At*HNL. Consequently, the substrates’ hydroxyl groups also interact differently with the polypeptide in the two enzymes, that is, they hydrogen bond to the serine residue of the catalytic triad (Ser80) and to a threonine residue (Thr11) in *Hb*HNL12a, 24 and to the catalytic His236 and the amide group of Asn12 in *At*HNL. All these differences appear to be mainly the result of two amino acid exchanges between the two enzymes, that is, Thr11 in *Hb*HNL is replaced by Asn12 in *At*HNL and Lys236 in *Hb*HNL is replaced by Met237 in *At*HNL. While the former difference in amino acids removes the positively charged interaction partner for the cyano group, the latter moves the position of the hydroxyl’s hydrogen bonding partner (hydroxyl of Thr11 vs. carboxamide of Asn12) by 3.6 Å, thereby creating a binding site for the hydroxyl group of the substrate near His236, and simultaneously opening the way for the cyano group to interact with the main-chain NH-groups of Phe82 and Ala13 (“oxyanion hole”, see below). In *Hb*HNL this site is blocked by Thr11, whose hydroxyl group is hydrogen bonded to the corresponding two amide nitrogen atoms.

Analysis of the molecular reasons for enantioselectivity is thus straightforward for each of the two enzymes, but the way inversion of enantioselectivity is effected for an identically folded protein by rather small changes in the vicinity of the active site is quite ingenious. Naturally occurring enantiocomplementary enzymes have been described for a number of enzyme classes, such as alcohol dehydrogenases^26^ or transaminases,^27^ although in some cases the enzymes display different structures as already described for HNLs. The abundance of enantiomeric natural products gives an idea, how many more enantiocomplementary enzymes are around but have not yet been discovered.^28^

Examples for structurally very similar enzymes with opposite enantiopreferences can be found among others in terpene cyclases,^29^ ketoreductases in polyketide synthases,^30^ sometimes even in the same organism like the *R*- and *S*-selective hydroxypropyl-thioethanesulfonate dehydrogenases from *Xanthobacter autotrophicus*.^31^ Furthermore, the enantioselectivity of several enzymes has been inverted by the introduction of a few amino acid changes through rational design or directed evolution.^32^ Only in rare cases, however, enantioselectivities comparable to the wild-type enzymes could be achieved.

Faber and Kazlauskas have tried to develop a classification system for enantiocomplementary enzymes based on active site structure.^33^ The *Hb*HNL/*At*HNL pair can best be classified in group 3—that is, same protein folding with exchanged locations of binding sites. However, as the active site of *At*HNL is not the exact mirror image of that of *Hb*HNL and the catalytic triad seems to be used in a slightly different manner (see below), a strict classification in this system appears difficult.

### Catalytic mechanism of *At*HNL

**Scheme 2:**
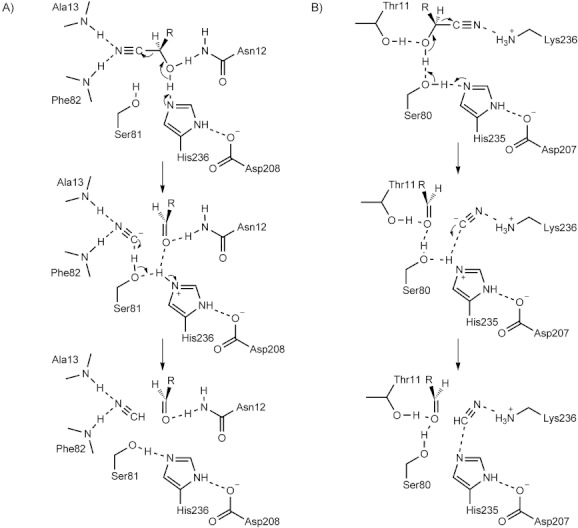
Proposed mechanisms for A) *At*HNL and B) *Hb*HNL.12a

Based on the modeled enzyme–substrate complexes, we propose a catalytic mechanism for *At*HNL shown in Scheme 2, where the *At*HNL mechanism is compared to the one established for *Hb*HNL.12a The cyanohydrin cleavage reaction is initiated by deprotonation of the cyanohydrin hydroxyl group by the catalytic triad residue His236. In *Hb*HNL, the hydroxyl group is bound to the catalytic triad residue Ser80 which acts as a mediator in the deprotonation by His235. In both cases, deprotonation is facilitated by hydrogen bonding interactions of the hydroxyl group with Asn12 (*At*HNL) or Thr11 (*Hb*HNL).

The second crucial element for HNL catalysis is electrostatic stabilization of the negative charge emerging at the cyano group in the course of the cleavage reaction.^13^ In *Hb*HNL this role has been assigned to Lys23612a and because of the close structural similarity this assignment is very likely also valid for the corresponding Lys237 in *Me*HNL. In *At*HNL this lysine is replaced by a methionine (Met237). In the absence of any other positively charged residue in the vicinity of the active site, electrostatic stabilization required for cyanohydrin cleavage has to be accomplished in a different way. We note that the cyano group lies in close proximity of and interacts with the backbone amide groups of Ala13 and Phe82 in the modeled *At*HNL complexes (Figure 2 A). This is indeed reminiscent of the so-called “oxyanion hole” in serine hydrolases (e.g., in the closely related esterase SABP2 from tobacco),^34^ where a negative charge emerging at the substrate’s carbonyl oxygen is stabilized by hydrogen bonding with two main-chain amide groups to facilitate formation of the acyl-enzyme intermediate. Additional stabilization might be provided by the dipole of the α-helix (from Phe82 to Ile93) following the “nucleophile elbow” in the α/β-hydrolase fold.^10^ These effects accumulate to a sufficiently positive electrostatic potential to stabilize the negative charge emerging at the cyano group.

The last step of the cyanohydrin cleavage reaction is protonation of the cyanide ion to yield hydrocyanic acid. In *At*HNL, this very likely is accomplished by His236 with the hydroxyl group of Ser81 as a mediator, whereas His235 directly protonates the cyanide in the *Hb*HNL catalyzed reaction. We note that the opposite chiralities of the substrates of *At*HNL and *Hb*HNL are paralleled by opposite routes of the abstracted proton: the proton is abstracted from the substrate hydroxyl by the histidine and returned via the serine to the cyanide in *At*HNL (clockwise), while it is abstracted from the substrate via serine and returned to the cyanide by histidine in *Hb*HNL (counterclockwise).

Mutagenesis data support our mechanistic proposal. The involvement of the *At*HNL catalytic triad residues (Ser81, His236 and Asp208) in catalysis was already confirmed by site-directed mutagenesis.^14^ In this study, we generated additional enzyme variants and tested them for activity (Table 2). As expected, the exchange of Asn12 by threonine (the residue observed at this topological position in *Hb*HNL) drastically decreased catalytic activity, presumably because the loss of one hydrogen bonding partner weakens substrate binding and prohibits the interaction of the cyano group with the “oxyanion hole”.

The overall structural and possibly mechanistic similarity of *Hb*HNL with α/β-hydrolases had already been noted when the first structure of this enzyme became available,^7^ lending support to the hypothesis that HNLs of this type have evolved from serine hydrolases. In fact, two amino acid substitutions in the active site of the esterase SABP2 from tobacco were sufficient to obtain weak HNL activity.^35^ These substitutions effected the introduction of a hydrogen bonding partner for the cyanohydrin hydroxyl group (Gly12 in SABP2 to Thr) and of a positively charged residue (Met237 to Lys). In terms of the evolution of HNL activity in general, the structure of *At*HNL indicates a closer relationship of this enzyme to α/β-hydrolases, as it appears to still utilize the “oxyanion hole” for electrostatic stabilization similar to the assumed predecessors.

## Conclusions

The crystal structure of *At*HNL revealed significant similarities to other HNLs from the α/β-hydrolase family. Analyses of modeled substrate complexes rationalize the observed *R* selectivity of *At*HNL and pinpoint crucial differences to other HNLs such as *Hb*HNL and *Me*HNL which explain the unexpected inversion of stereoselectivity. The proposed mechanism contains all elements considered crucial for HNLs, that is, the existence of a general base and the stabilization of the negative charge emerging on the cyano group upon C–C bond cleavage. In contrast to *Hb*HNL (and *Me*HNL), however, the latter is not accomplished by a positively charged (lysine) residue but presumably by a combination of main-chain amide hydrogen bonds and a helix dipole more similar to the stabilization of the oxyanion in α/β-hydrolases.

## Experimental Section

**Crystal structure analysis:** *At*HNL was expressed in *E. coli* BL21(DE3) as previously described.^14^ The enzyme was purified from the crude cell extract using standard column chromatography techniques (ion exchange and size exclusion). Samples used for the crystallization trials contained the enzyme at a concentration of 20 mg mL^−1^ in acetate buffer (10 mm, pH 6). The protein concentration was determined according to Bradford’s method.^36^ Diffraction quality crystals were obtained using sitting-drop vapor diffusion with reservoir solutions consisting of PEG-3350 (10–18 % *w*/*v*) in BisTris buffer (100 mm, pH 6).

Before flash cooling in liquid nitrogen, crystals were soaked for about 30 seconds in a solution consisting of the reservoir solution containing glycerol (25 % *v*/*v*) for cryoprotection. A diffraction data set extending to 2.5 Å resolution was collected at cryogenic temperatures using synchrotron radiation at the EMBL beamline X13 at the DESY in Hamburg. Data reduction involved the programs DENZO and SCALEPACK^37^ as well as software from the CCP4 suite.^38^ At first, the data were processed as orthorhombic *C*222_1_ (*a*=63.57 Å, *b*=77.77 Å, *c*=223.28 Å), but statistical analyses of the obtained structure factor amplitudes indicated the presence of significant twinning. The data were thus reprocessed in the monoclinic spacegroup *P*2_1_ (*a*=50.25 Å, *b*=223.31 Å, *c*=50.20 Å, *β*=101.47°) and the twinning transformation (*l*, −*k*, *h*) was identified. For structure solution the data were detwinned using the program DETWIN from the CCP4 suite,^38^ assuming a twinning fraction of 0.448.

A homology model of *At*HNL was built using the program MODELLER 8v1,^39^ based on structures of the HNLs from *H. brasiliensis* (*Hb*HNL, PDB ID: 1QJ4) and *M. esculenta* (*Me*HNL, PDB ID: 1DWP) as well as of the salicylic-acid-binding protein from tobacco (PDB ID: 1XKL) as templates. These proteins share sequence identities between 44 and 49 % with *At*HNL. Molecular replacement using PHASER^40^ yielded an unequivocal solution with four protein molecules in the asymmetric unit. The structure was refined using PHENIX^41^ against the original twinned data yielding a final refined value for the twinning fraction of 0.481. Model building and fitting steps involved the graphics program COOT^42^ using *σ*_A_-weighted 2*F*_o_−*F*_c_ and *F*_o_−*F*_c_ electron density maps.^43^ *R*_free_ values^44^ were computed from 5 % randomly chosen reflections not used for refinement. Special care was taken that reflections related by the twinning transformation were both contained in the test set. Noncrystallographic symmetry (NCS) restraints were applied throughout the refinement. A total of 68 well-defined water molecules and a chlorine ion were included into the model. In all four chains, the first two N-terminal residues were not visible in the electron density, in two chains the last C-terminal residue was missing. A Ramachandran plot shows almost all residues in the core and allowed regions with the exception of Ser81, which was observed in the disallowed region in all four chains. This residue is located in the so called “nucleophile elbow” which is known to require a somewhat strained main-chain conformation in α/β-hydrolases.^10^ Details of the data collection, processing and structure refinement are summarized in Table 1. The atomic coordinates and structure factors have been deposited in the Protein Data Bank (PDB) under ID 3DQZ.

**Modeling of substrate complexes:** Of the four crystallographically independent *At*HNL molecules the one with the lowest average *B* factor was chosen for the docking calculations using AutoDock v4.^21^ Aspartate, glutamate, arginine and lysine residues were treated as charged, protonation and tautomeric states of histidine residues were chosen in order to optimize hydrogen bonding interactions with surrounding residues. Molecular models of (*R*)- and (*S*)-mandelonitrile were built and optimized using the program Sybyl v6.8 (Tripos Inc.). During the docking simulations the protein was kept rigid, and the position and orientation of the substrates as well as two torsion angles (for the phenyl and the hydroxyl group) were allowed to vary. A hybrid genetic algorithm with phenotypic local search designated as a Lamarckian genetic algorithm^21^ was applied in 50 independent simulations with populations consisting of 300 random structures and a maximum of 300 generations. The best individual of each generation automatically survived, the mutation and crossover rates were set to 0.02 and 0.80 respectively. The probability for performing a local search (up to 300 iterations) was 10 %. A cluster analysis with an RMSD-cutoff of 1.0 Å was performed. The resulting complex structures were further optimized by molecular mechanics using AMBER v9.^45^

**Introduction of point mutations:** Point mutations were introduced using the QuikChange PCR protocol from Stratagene (QuikChange II site-directed mutagenesis kit). For amplification, Pfu-Turbo polymerase from Stratagene was employed; p*Athnl*, containing the *At*HNL-gene in the vector pET28a (Novagen) was used as a template with the following primer pairs (with the underlined codon representing the changed position):

Asn12Thr, 5′-CGTGTT AGTTCA CACCGC TTATCA TGGAGC, 3′-GCTCCA TGATAA GCGGTG TGAACT AACACG;

Met237Lys, 5′-GGCGGA GATCAC AAAGTG ATGCTC TCCAAA CC, 3′-GGTT TGGAGA GCATCA CTTTGT GATCTC CGCC;

Met237Leu, 5′-GGCG GAGATC ACCTGG TGATGC TCTCCA AACC, 3′-GGTT TGGAGA GCATCA CCAGGT GATCTC CGCC.

Introduction of the expected mutation was verified by plasmid sequencing.

## Supplementary material

Detailed facts of importance to specialist readers are published as ”Supporting Information”. Such documents are peer-reviewed, but not copy-edited or typeset. They are made available as submitted by the authors.
