# Supplementary material for: Hydroxynitrile Lyases with α/β-Hydrolase Fold: Two Enzymes with Almost Identical 3D Structures but Opposite Enantioselectivities and Different Reaction Mechanisms
Source: Chembiochem. 2012 Jul 31;13(13):1932–9. doi: 10.1002/cbic.201200239 (PMC3444685; doi:10.1002/cbic.201200239)
Supplement: Supplementary file 1 [file cbic0013-1932-SD1.pdf]

CHEMBIOCHEM

Supporting Information

© Copyright Wiley-VCH Verlag GmbH & Co. KGaA, 69451 Weinheim, 2012

Hydroxynitrile Lyases with α/β-Hydrolase Fold: Two Enzymes with Almost Identical 3D Structures but Opposite Enantioselectivities and Different Reaction Mechanisms

Jennifer N. Andexer,[a] Nicole Staunig,[b] Thorsten Eggert,[c] Christoph Kratky,[b] Martina Pohl,[d] and Karl Gruber*[b]

cbic_201200239_sm_miscellaneous_information.pdf

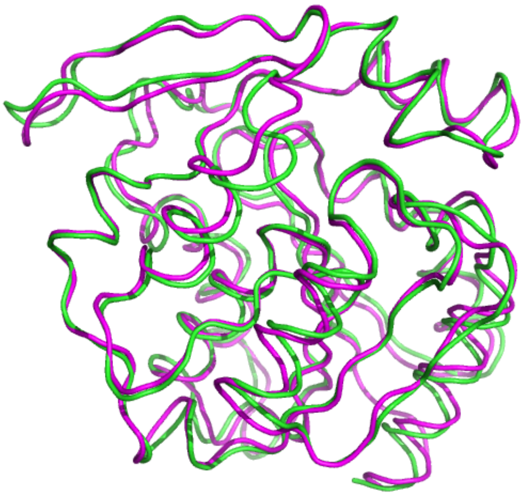

Figure S1: Superposition of the homology model of *At*HNL[14] (green) and the present crystal structure of the enzyme (magenta). The root-mean-square-deviation (rmsd) was 0.9 Å for 243 superimposed Cα-atoms. The figure was prepared using the program PyMOL (http://www.pymol.org).

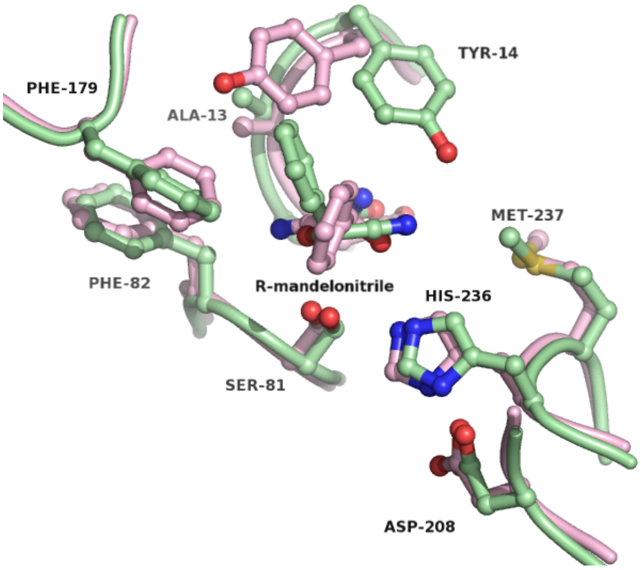

Figure S2: Modeled complexes of *At*HNL and (*R*)-mandelonitrile. The first model (green) was built into the homology of *At*HNL[14] and was based on the crystal structure of *Hb*HNL in complex with (*S*)-mandelonitrile.[23] The model presented in this paper (pink) was generated using the crystal structure of *At*HNL and an unbiased docking approach.
